# Anatomy and Physiology of Dentine

**Published:** 1853-01

**Authors:** J. De Haven White


### ARTICLE IV.

*Anatomy and Physiology of Dentine, an Essay read before the American Society of Dental Surgeons, convened at Newport, R. I., August,* 1852. By J. DE HAVEN WHITE, M. D., D. D. S.

MR. PRESIDENT AND GENTLEMEN:—The subject which I have chosen for the present essay is the *Anatomy and Physiology of Dentine.* Anatomists are in the habit of dividing a tooth into three parts, viz. A *body*, a *neck* and a *root.* The body is that portion which appears above the gum. It is protected by a semi-transparent crystalline and insensible substance, the *enamel.* The neck is embraced by the gum; is about a line in breadth, and intervenes between the cessation of the enamel and the margin of the alveolus. The remaining part is the root, which articulates by the *gomphosis* articulation, with the alveolar process. There are three different tissues entering into the composition of the structure of a tooth; two of these may be considered peculiar to this organ, viz. The dentine or tooth substance which forms the body and root, and the enamel, which invests the body of the tooth. The third tissue, termed *cementum*, and by some *secondary dentine*, forms a thin layer, which invests the root of the tooth, from its neck to the extremity of its fang, where it is thicker than at the neck, and as age advances, it is deposited upon the surface of the dental cavity. This deposit commences at the upper part of that cavity, and gradually increases with the years of the individual. In very old persons it so completely fills up the dental cavity, as to leave behind no rudiment of its previous existence.

The arteries which supply the teeth, have their origin from the *internal maxillary* branch of the *external carotid*, and the veins, by which the blood is returned from the teeth, after following the course of the arteries, from the *internal maxillary vein*, which terminates in the *external jugular*.

The nerves which supply the teeth with general sensibility, are the *fifth cerebral nerves*, commonly called the *fifth pair*. Every tooth has within its body a cavity, called the *dental cavity*, which is very similar in shape to the class of teeth it occupies, being larger in the body, and narrowing off as the root diminishes, terminating in a capillary foramen at the extremity of the root. This foramen gives passage to the blood-vessels and nerves composing the *dental pulp*. This pulpy substance is enveloped with an exceedingly delicate and vascular membrane, which is a reflection of a similar membrane, covering the external surface of the root, which membrane is also the production of a membrane called the *periosteum* of the *maxillary bone*. There are, therefore, 1st: The maxillary periosteum, which lines the socket of the tooth; 2d, an external periosteum of the root; and 3d, an internal periosteum, lining the dental cavity; the two latter membranes in health, perform similar functions, namely, that of nourishing the bony substance of the tooth.

*Structure of Dentine.*—According to recent microscopical observations, dentine or tooth substance is composed of two distinct parts, "*dentinal tubes*" and a uniting medium—an intertubular tissue. The tubes have distinct parietes, which nearly or quite equal their calibre, and although usually empty, yet sometimes, even in healthy dentine, appear to contain a minutely granulated substance. Their arrangement is radiated, the centre of radiation being the pulp-cavity.*

"Each tube commences on and contributes its share to form the walls of the pulp-cavity, from which point it advances in an

* Tomes.

*undulating* course towards the surface of the tooth; the general direction being nearly rectangular with the surface from which it started. If a single tube be traced through its whole length, it will be found to have made two or three large bends, and in addition to these, which are called the primary curves, a vast number of small undulations; these latter are termed the secondary curves."

"In their course the dentinal tubes give off branches, and these, meeting with others of similar character, anastomose, and thus form frequent connections throughout the whole substance of the tooth. The nearer the tubes approach the enamel, or the cementum, the more frequent are their branchings, till at last they terminate either by anastomoses with adjoining tubes, or pass into the external structures, or else terminate in a dilatation—or become so extremely minute, that they are lost to the sight."

The foregoing description may be considered a general outline of the dentinal tubes, subject, however, to many modifications in different parts of the tooth, as for instance: "The coronal tubuli suffer but little diminution in size, till they divide, and then the branches conjointly are larger than the parent tube. Sometimes, indeed, two tubes unite near the enamel, and from the larger tube so formed, two or three minute ones are given off." And the terminal branches, having arrived at the line of junction between the dentine and enamel, are lost, or recurve and anastomose with contiguous tubes, or pass across the line of junction into the enamel, or else end in a dilated extremity near the surface of the dentine. "In the tubuli which occupy the neck of the tooth, instead of pursuing an uninterrupted course, till within a very short distance of the termination of the dentine," and then dividing into secondary branches, like those of the crown; the tubes of the neck give off during the latter third of their course, numerous fine hair-like branches, visible only under a very high magnifying power." "The parent tube at the same time, gradually decreases in size till it is no longer traceable." In the fangs of the teeth, the anastomosis between the tubes is far more general and frequent than in the crown, and their distribution much less regular. "There is considerable discrepancy with regard to the size of the dentinal tubes between different observers; some estimating them at from the $\frac{1}{10000}$ to the $\frac{1}{4620}$ of an inch in diameter. That there is a great difference in different specimens, and even in different parts of the same specimen, there is not the slightest doubt. At present, the dentinal tubes and cells, and the pulp-cavity, have alone been described as existing in the dentine; but, in addition to these we have, in many instances, canals for vessels traversing the tissue, just as we have the Haversian canals perforating bone."*

Frequently after extracting a tooth, I have enlarged the foramen at the apex of the fang, with a drill, sufficiently to insert a strong metallic plate, and then upon forcing an instrument into the pulp-cavity, the blood contained in it would be observed to escape from numerous pores over the fang of the tooth, proving that there are more than one point of entrance and exit for blood vessels in the root of a tooth, call them what you may. "The dentine is, in some animals, in all cases vascular; the teeth of the walrus offer an example, as do those of the kangaroo and the rabbit." The structure of the enamel is very simple, it is composed of semi-transparent fibres, placed side by side and closely united. Their form is an approximation to a six-sided prism, and tolerably uniform, being from the $\frac{2}{10000}$th to the $\frac{3}{10000}$th of an inch in diameter, and are held together by a membrane, called enamel membrane. "The cementum is considered, by different observers, as being similar to osseous tissue,† and highly vascular."

The foregoing hasty and limited description of the tooth and its structure, will be considered sufficient to understand its *physiological* condition, as is observed by Mr. Tomes: "That osseous and dental tissues are, in form and arrangement of their cells and tubes, very closely allied. But the relations of their ultimate tissues are yet closer; for dentine and cementum, and

* Tomes.

† Tomes, Goodsir and others.

probably enamel, are built up like bone, of more or less spherical granules, the difference in the tissues being in the arrangement of the granules, and in the relative quantity of earthy matter with which they are impregnated." Chemical analysis shows a very close alliance between their organic substances, 28 per cent. in dentine, and in bone 33.

It is clearly established that the dentine is tubular in its structure, and that it receives a supply of nutrition in consequence, there can be but little doubt. I have very frequently broken open numbers of teeth after extraction, and taken out the pulps, weighed them, and exposed them to a heat ranging from 90 to 100 degrees for several days, and then, on weighing them again, find that teeth from young persons, say 20 years of age, in 100 grs. lose 16 grs., and one batch of 100 grs. lost 12, from older individuals; and upon soaking in common water for one week, gained 10 grs. again; taking advantage of this fact, I placed a number of teeth in the tincture of red sanders, some after being dried, and some before, and in a few months they became tinged with coloring matter, as the accompanying specimens will show. I have referred to this latter experiment in a paper which I had the honor of reading before the Pennsylvania Society of Surgeon Dentists, in June, 1846, and since published by order of the society in Stockton's Dental Intelligencer, and some of these specimens which have been in the tincture for years, are so highly tinged, that were it not for the shape of the tooth, it would scarcely be detected as tooth substance. That the coloring matter can enter as well from the external surface of the tooth, is proven in the following manner: take a perfectly sound tooth, drill the foramen at the apex of the root sufficiently large to receive a strong metallic plug, and then place it into the tincture, and it will become highly tinged also, but not in as short a time as if the dental cavity were also exposed to the action of the fluid. I believe I have the honor to be the first to have obtained the complete infiltration of the dentine with coloring matter of a completely formed tooth out of the body. This experiment explains the cause of the blueness of the teeth after the destruction of the pulp; the decomposed pulp is absorbed, or when it disappears, the foreign substances which enter the dental cavity, through the cavity of decay, are also absorbed; it also proves the injurious consequences of a too frequent and too long an application of poisons to teeth to destroy the pulp. In treating tooth-ache, the poisons are absorbed by the tubuli, and are brought in contact with the external membranes, and produces inflammation. The accompanying specimen shows the enamel to be slightly susceptible to the effects of coloring matter also, and the extremities of the fangs less so than the body of the tooth. That the teeth could not be imbued with coloring matter when once formed, has been, heretofore, held as conclusive evidence that the tooth substance was devoid of vascularity, by the most eminent writers—*Hunter*, *Horner*, and many others. The experiments of Mr. Hunter, of feeding growing animals upon a colored diet, with which you are doubtless familiar, producing a layer of discolored tissue, without affecting that already formed, and which experiments, Professor Horner asserts, were verified by his own observation, proving that the tissue already formed is not vascular, is by no means satisfactory to my mind; they merely prove that the teeth are more susceptible of being tinged with coloring matter while forming, than when once formed. Neither is a suspension of the colored diet for a few weeks, in order to produce a layer of white and a layer of red, (which twelve months, perhaps, would wholly obliterate,) conclusive. The time, "a few weeks," in so dense a tissue, where the circulation is feeble, is not sufficient to change wholly its character. Again, the coloring matter must be a foreign substance; and that it remains there, does not prove that a "commutation of nutritious particles" is not constantly going on around it; for, if we reflect, that coloring matter of various hues and shades, in the form of the well known India ink, is constantly being introduced into the skin, where we are quite sure a very rapid change of "nutritious particles" is going on, at all times, and does not remove during a long life, the most delicate devices, which are frequently made there, the whole argument falls at once to the ground. It is asserted that the red blood globules are too large to enter the dentinal tubes, as the average size of a blood globule is $\frac{1}{3000}$th of an inch in diameter, and the dentinal tubes $\frac{1}{4620}$th. It is well known by dental practitioners, that if the dental pulp be partially destroyed by some corrosive agent, that the violent inflammatory action established thereby, induces a red tinge in the body of the tooth, or in a single root, as the case may be, in which the inflammatory action would seem to have been greatest. The accompanying specimen will clearly illustrate this fact; it is a case where arsenious acid was applied for destroying the pulp, but as the tooth excited considerable pain some days after, it was extracted, when its present appearance was observed. It will be seen that the root most discolored, is the one opposite the point of the exposure of the pulp, and that the one most distant, the palatine, is of the natural appearance. Injuries of the dental pulp without the application of any corrosive agent, will induce the same appearance of the tooth substance. I have proven by repeated experiment, that to excite bleeding of the pulp, and then plug the cavity of decay, that the whole tooth substance will become tinged with red; whether it is by the passage of the blood globules entire, or merely by the infiltration of their coloring matter, after they are disintegrated, I am not able to decide; but whether the tubuli are too small to receive blood globules or not, does not, to my mind, seem of great physiological importance; but that they have not the "liquor sanguinous" circulating through them, as other white tissues have, is of great moment. To prove this point, I will relate one single case, which I observed about seven years ago. A Mr. T., a most intelligent gentleman of our city, called upon me to have a front tooth plugged which was very much decayed; it was of very large size, and translucent, and very nearly decayed to the nerve. After having removed all the decayed matter from the tooth, and in looking carefully to see whether the pulp was exposed or not, I observed a dampness in the bottom of the cavity, which I wiped away, but which returned again as often as wiped away; seeing that it was oozing from the pulp cavity, I waited for a few minutes, and as much as a half drop accumulated in the cavity. I did not plug the tooth under such circumstances, but applied to the part some tannic acid, and in a few days the oozing ceased entirely, when I plugged the tooth as firmly as possible with gold; but in a few months I observed the tooth turning blue opposite the pulp cavity beneath the plug, which blueness spread from the centre of the tooth towards its surface, until it became very much discolored; the blueness spread from within out, and not from without in, as is the case when the plug is not solid; besides the tubal character of the tooth substance, there is another condition of it which will, perhaps, more fully establish its organization; I mean the extreme sensibility of the dentine to the touch, and when acted upon by chemical agents, for which various explanations have been advanced with regard to the pain excited, I think but one explanation can be given, namely, that of *irritation by actual contact with nervous tissue.* With relation to pain by friction, or to the touch, some contend that it is due to the tubuli being filled with a light serum, making a kind of column, the base of which, resting upon the dental pulp, renders it (the dental pulp) sensible of any impression which may be made upon its apex, which apex terminates beneath the enamel. I have, in many instances, endeavored to render this view of the case practical, by applying a heated instrument to a sensitive surface, believing that if the explanation were true, it could be rendered useful by drying out the extremities of the tubes, and thereby lessen sensibility, but it has failed. Others allege that it is in consequence of the friction or concussion of solids against the dense fibres of which dentine is composed, being communicated along them until it reaches the dental pulp and periosteal membranes; again, others assert that it is the result of the actual contact of substances with nervous fibre, which is distributed in the dentine. The latter explanation seems the most plausible, which the following simple experiment will fully prove. Place an escharotic in contact with the exposed and sensitive dentine, no matter how distant it may be from the pulp, and leave it there for a time, longer or shorter, as the case may be, remove it, and all sensibility will have gone from the surface in which it was in contact; but cut down a little of the surface, and you will again find sensitive substance; for instance: place a small quantity of arsenious acid immediately beneath the enamel, and secure it with some white wax for a few hours, and it will destroy all feeling on the surface; but leave it a longer time and it will be absorbed by the tubuli and destroy the sensibility of the whole tooth substance, when it is clear that it only acts upon the organic part of the tooth, leaving the calcarious fibres as capable of communicating friction or concussion, as before; if the structure of the tooth were at all broken up, by any such application, according to the first explanation, it would only render it more sensitive, as a greater quantity of fluid would then be absorbed.

Another circumstance which bears strongly in favor of the vascularity of dentine is, that during the absorption of the roots of the deciduous teeth, if the crown should be held in its place by the adjoining teeth, or should not be knocked loose by chewing, when its roots were absorbed, that the pulp which had its share in absorbing the roots, now lays hold of the crown, after the roots have been absorbed, and carries the whole of its dentinal substance away, also; a circumstance which could not be explained if the blood vessels stopped against the walls of the dental cavity, but, on the contrary, pass through to the enamel membrane; and again, under such circumstance, it requires a degree of force applied to the pulp to pull it away from its contact with the dentine; showing that it dips its vessels down into this substance. I have a specimen of this absorption with me. This peculiar absorption is not only confined to the deciduous teeth, but occurs to the adult teeth also; although it may be induced in the adult teeth by a different exiting cause than is concerned in the former class of teeth. Doubtless all present have plugged teeth when they believed that the pulp was not quite exposed, but in time pain would set in, or they would find from discoloration of the tooth, that the pulp had become dead, or inflamed; and on removing the pulp there would be a distinct opening from the cavity of decay to the pulp-cavity, so large that if such opening had existed before the plugging had been done, it would have been observed. It would seem from this circumstance also, that the deposition of cementum, which is looked for to take place beneath a plug, where it nearly reaches the pulp, is very uncertain. If irritation is excited at all by a close proximity of decay to the pulp, it is as likely to provoke absorption as deposition of the cementum or dentine.

In conclusion, in the language of a recent writer, (Mr. Tomes,) "Strictly speaking, all tissues are in themselves extra vascular, that vessels do not permeate their substance, but pass only between their fibres, lamina, or granules, whatever be the structure of the tissues." "Taking the relative frequency of vessels in a tissue, as an index of the degree of its organization, teeth will be placed near the bottom of the scale, but different grades will be assigned to their three component tissues." "The cement, or tooth-bone, will occupy the first; the dentine the second; and the enamel the third." It is a law of nature, that in passing from one form of organized matter to another, no sudden transition shall be made, but that the individual changes shall be so gradual as to be almost imperceptible. This law we find beautifully exemplified in the gradual change of structure in passing from the *cement* to the *dentine*, and from the latter to the *enamel*.

